# BN Embedded Polycyclic π-Conjugated Systems: Synthesis, Optoelectronic Properties, and Photovoltaic Applications

**DOI:** 10.3389/fchem.2018.00341

**Published:** 2018-08-07

**Authors:** Jianhua Huang, Yuqing Li

**Affiliations:** College of Materials Science and Engineering, Huaqiao University, Xiamen, China

**Keywords:** BN-embedded unit, isoelectronic structure, π-conjugated material, organic solar cell, device performance

## Abstract

In the periodic table of elements, boron (B, atomic number, 5) and nitrogen (N, atomic number, 7) are neighboring to the carbon (C, atomic number, 6). Thus, the total electronic number of two carbons (12) is equal to the electronic sum of one boron (5) and one nitrogen (7). Accordingly, replacing two carbons with one boron and one nitrogen in a π-conjugated structure gives an isoelectronic system, i.e., the BN perturbed π-conjugated system, comparing to their all-carbon analogs. The BN embedded π-conjugated systems have unique properties, e.g., optical absorption, emission, energy levels, bandgaps, and packing order in contrast to their all-carbon analogs and have been intensively studied in terms of novel synthesis, photophysical characterizations, and electronic applications in recent years. In this review, we try to summarize the synthesis methods, optoelectronic properties, and progress in organic photovoltaic (OPV) applications of the representative BN embedded polycyclic π-conjugated systems. Firstly, the narrative will be commenced with a general introduction to the BN units, i.e., B←N coordination bond, B-N covalent bond, and N-B←N group. Then, the representative synthesis strategies toward π-conjugated systems containing B←N coordination bond, B-N covalent bond, and N-B←N group will be summarized. Afterwards, the frontier orbital energy levels, optical absorption, packing order in solid state, charge transportation ability, and photovoltaic performances of typical BN embedded π-conjugated systems will be discussed. Finally, a prospect will be proposed on the OPV materials of BN doped π-conjugated systems, especially their potential applications to the small molecules organic solar cells.

**Figure:**
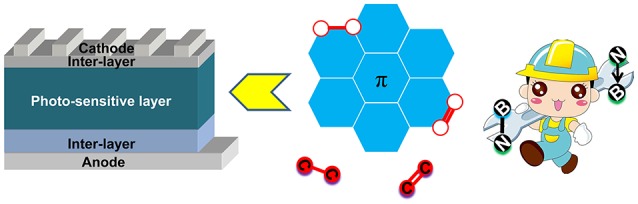
**GRAPHICAL ABSTRACT** Design motif of BN embedded Pi-units for photovoltaic application.

## Introduction

**Figure 1:**
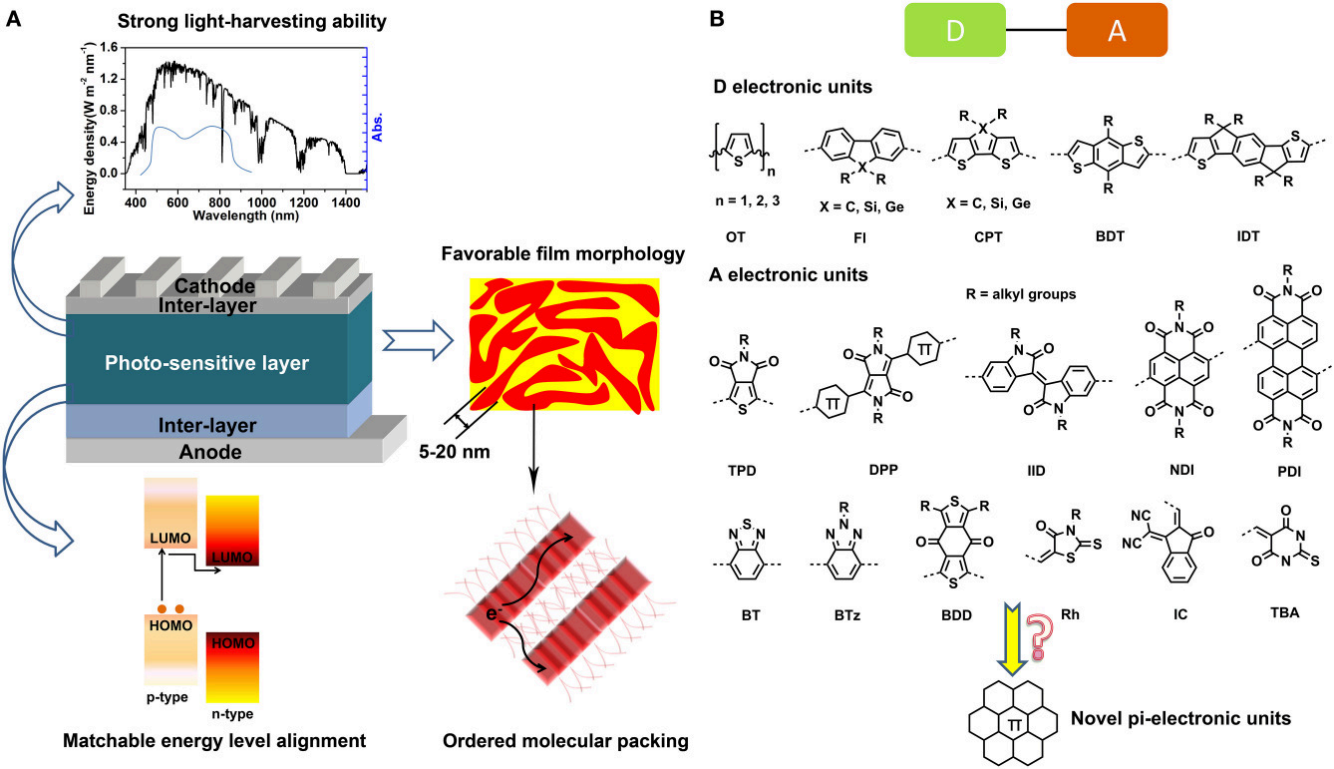
Requirements for high-performance OPV devices **(A)** and representative D and A π-electronic units **(B)**.

The past years have witnessed a fruitful advance of organic conjugated materials and great enthusiasm was fueled to develop novel π-molecules and judiciously apply them to organic electronic devices, e.g., organic field effect transistors (OFETs) (Gsänger et al., 2016; Li M. et al., 2018), organic light emitting diodes (OLEDs) (Grimsdale et al., 2009), organic solar cells (OSCs) (Lu et al., 2015; Zhan and Yao, 2016), organic thermoelectric devices (OTEDs) (Shi et al., 2015; Huang et al., 2016; Lim et al., 2018), and organic photodetectors (OTDs) (Wang et al., 2016a; Benavides et al., 2018; Murto et al., 2018). Especially, the bulk-heterojunction (BHJ)-type OSCs adopting organic semiconductors as photo-sensitive layers have been considered a promising candidate for the next generation of green energy due to solution processability, low cost, light weight, flexibility features of organic materials. The photo-sensitive layers of OSCs are blends of an electron-donor (p-type) and an electron-acceptor (n-type) with nano-phase separated morphology. Although the fullerene derivatives, e.g., phenyl-C_61_-butyric acid methyl ester (PC_61_BM) and phenyl-C_71_-butyric acid methyl ester (PC_71_BM) have been the dominant acceptor materials in a long time (Sariciftci et al., 1992), both the electron-donor and electron-acceptor materials have been extended to π-conjugated linear molecules, star molecules, oligomers, and polymers in recent years. To achieve satisfactory power conversion efficiency (PCE), the photo-sensitive materials should be featured by the following points (Figure 1A), (1) strong light-harvesting ability resulting from wide absorption band and strong absorption coefficients; (2) appropriate energy level alignment between the p-type and n-type materials to ensure efficient built-in field and driving force for exciton dissociation; (3) proper aggregation and crystallization ability for both of p-type and n-type materials to form well-defined blend film with desirable micro-morphology, e.g., domain sizes and molecular stacking order; (4) fairly well-charge carrier mobility, i.e., electron and hole mobility to facilitate the charge transportation and collection. These features are closely related to the material properties and device preparation technics. Thanks to the continuing devotion on material design and device optimization, the PCE of single junction photovoltaic devices based on organic semiconductors have been promoted from the initial 1% in 1986 (Tang, 1986) to 10–13% recently (Gupta et al., 2013; Chen et al., 2015; Zhang Z. G. et al., 2017; Li W. et al., 2018), illuminating the bright future of OSCs for low-cost and portable energy provision. However, in contrast to the inorganic and hybrid photovoltaics, for example, the silicon solar cells and perovskite solar cells, whose efficiencies are commonly on the magnitude of ca. 20% (Sun, 2015; Meng et al., 2016), the OSCs have a large offset to promotion. In fact, theoretical models based on Shockley–Queisser detailed balance approach predicted a reachable PCE of 20–24% for OSCs (Janssen and Nelson, 2013), Moreover, in the current stage, excellent photovoltaic materials capable of accomplishing efficiencies higher than 10% are limited. As such, large amount of fundamental explorations on developing novel photovoltaic materials are required to thrust the overall progress of OSCs.

The most popular strategy to construct the organic photovoltaic materials involves the covalently bonding of various conjugated units with electron-rich (D) or electron-deficient (A) nature to obtain D-A type linear molecules, star molecules, oligomers, and polymers. These D and A π-electronic units are basic building blockings that critically determine the optoelectronic properties and photovoltaic performances of the photovoltaic materials. Consequently, the design and structural tailoring of π-electronic units are essential for the construction of photovoltaic materials. To now, outstanding D units such as oligothiophene (OT), fluorene (Fl), cyclopentadithiophene (CPT), benzodithiophene, (BDT), and indacenodithiophene (IDT) and A units including perylene diimide (PDI), naphthalene diimide (NDI), diketopyrrolopyrrole (DPP), isoindigo (IID), thieno[3,4-c]pyrrole-4,6-dione (TPD), benzothiadiazole (BT), benzotriazole (BTz), benzo[1,2-c:4,5-c′]dithiophene-4,8-dione (BDD), rhodanine (Rh), cyano indone (IC), and N,N′-diethyl thiobarbituric acid (TBA) were revealed in literature (Figure 1B) (Lu et al., 2015; Zhan and Yao, 2016). Additionally, classic dye molecules such as phthalocyanine (Pc), porphyrin (Pr), and squaraine (SQ) are also frequently reported for construction of OPV materials (Chen et al., 2012, 2014; Chen G. et al., 2015). Developing novel D or A π-electronic units has being an energetic realm. A typical strategy of introducing heteroatoms including O, N, P, S, Se, Si, Ge, and B, etc. into the polycyclic aromatic hydrocarbons (PAH) backbone is widely used to tailor the properties of π-electronic units (Stepien et al., 2017).

**Figure 2:**
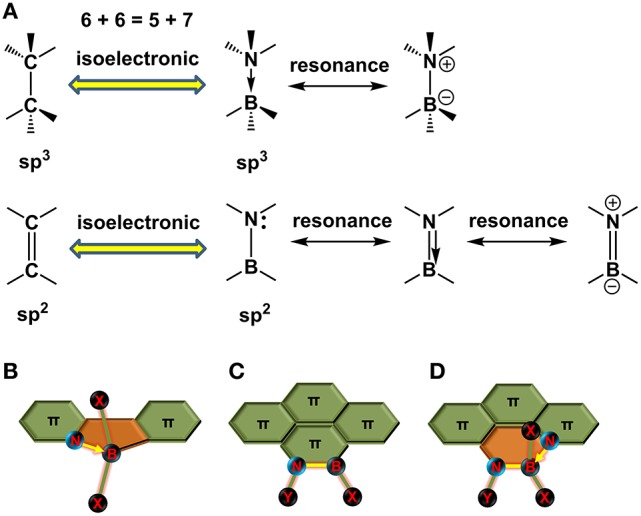
Isoelectronic structures of CC and BN units **(A)** and schematic diagram of π-electronic units containing B←N coordination bond **(B)**, B-N covalent bond **(C)**, and N-B←N group **(D)**. X, Y, and Z represent the halogen atoms, alkyl chains, or phenyl groups.

When one gives a glance to the periodic table of elements, it's a cinch to perceive the neighbor elements of carbon (C, atomic number, 6), i.e., boron (B, atomic number, 5) and nitrogen (N, atomic number, 7). Thus, the total electronic number of two carbons (12) is equal to the electronic sum of one boron (5) and one nitrogen (7). Accordingly, replacing two carbons with one boron and one nitrogen in a π-conjugated structure gives an isoelectronic system, i.e., the BN embedded π-conjugated system, in contrast to its all-carbon analogs. The bonds between the B and N can be formed as coordinated bond (B←N) and covalent bond (B-N), corresponding to the isoelectronic units of C–C and C = C (Figure 2A), respectively. Replacing CC unit with BN unit in the conjugated skeleton is favorable for property adjustment. On the one hand, the BN would alter the electronic nature of the conjugated backbone due to the different electron-negativity of heteroatoms with that of carbon atom. On the other hand, the BN also enhances the dipolarity of hydrocarbon skeletons and thus boost the inter-molecular interactions. Additionally, replacing carbons with BN usually maintains the good co-planarity and rigidity of the backbones. All these features are desirable to design novel π-electronic units for photovoltaic materials construction. Taking the isoelectronic compounds of ethane and ammonia-borane (NH_3_←BH_3_, AB) for an example, ethane is gaseous at ambient temperature with a dipole moment of zero and weak inter-molecular interactions (Pritchard and Kern, 1969), whereas AB is a solid state at room temperature with a strong dipole moment of 5.2 D and inter-molecular BH…NH interactions (Leroy et al., 1993). For B←N embedded aromatic systems, the first report was in 1963 by Morrison et al. (Letsinger and MacLean, 1963). Recently, a series of conjugated materials containing B←N bonds have been revealed for OSCs application (Dou et al., 2015, 2017). For the B-N covalent bond embedded aromatic structures, the research history has been almost one century since the first synthesis of borazine in 1926 (Stock and Pohland, 1926). In 1950s and 1960s, Dewar and coworkers conducted pioneering work on synthesis of BN doped PAH (Dewar et al., 1958; Dewar and Dietz, 1959; Chissick et al., 1960; Davies et al., 1967). Since then, little progress in this field has been made due to limited characterization means at that time. Recently, the B-N embedded polycyclic aromatic systems are experiencing a renaissance with fast development of synthesis protocols and widely application to H_2_ storage, OLEDs, and OFETs (Jaska et al., 2006, 2007; Bosdet et al., 2007a; Liu and Marder, 2008; Campbell et al., 2012; Hashimoto et al., 2014; Wang et al., 2015a,b, 2016b; Beniwal et al., 2017; Ishibashi et al., 2017). Additionally, N–B←N group, the comprehensive form of B←N coordination and B-N covalent bonds, also widely appears in the conjugated units, such as BODIPY (Loudet and Burgess, 2007). All of these BN perturbed structures have unique properties and are intensively interested in terms of synthesis routes, optoelectronic properties, and electronic device performances. However, studies on the photovoltaic applications of BN embedded π-electronic units are still in infancy. In this review, we are going to summarize the synthesis routes toward π-electronic units containing B←N coordination bond, B–N covalent bond, and N–B←N group (Figures 2B–D), and discuss their optoelectronic properties, as well as their applications in photovoltaic devices.

## Synthesis routes

### Synthesis of π-electronic units containing B←N coordination bond

#### Alkyl lithium (e.g., n-BuLi)/ aryl boron (e.g., BPh_3_) system

**Figure 3:**
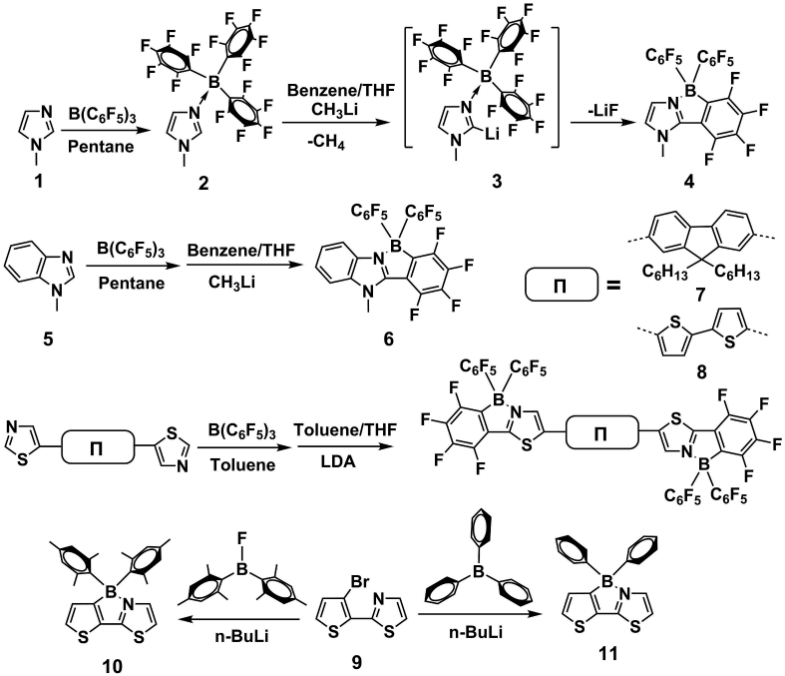
Examples of synthesis routes toward B←N embedded units involving alkyl lithium / aryl boron reaction system.

In 2002, Erker et al. reported an intramolecular nucleophilic aromatic substitution reaction, using CH_3_Li and B(C_6_F_5_)_3_ to prepare the tricyclic fused structures containing B←N coordination (Dominik et al., 2002). As shown in Figure 3, the starting *N*-Methylimidazole **1** was coordinated with strong Lewis acid of B(C_6_F_5_)_3_ to form adduct **2**. Deprotonation at the C-2 position of imidazole heterocycle was accomplished by treatment with CH_3_Li, affording intermediate **3**, which experienced a rapid intramolecular nucleophilic aromatic substitution reaction with one of the adjacent C_6_F_5_ groups to generate fused π-electron unit **4**, containing B←N coordination bond. One year later, they replaced the starting reactant with 1-methylbenzimidazole **5**. Using the same routes, larger conjugated π-electron unit **6** was obtained (Vagedes et al., 2003). Similarly, in 2010, B←N perturbed structures with further extended conjugation (**7** and **8**) were synthesized using the same strategy (Job et al., 2010). In 2006, Yamaguchi et al. reported the synthesis of π-electron systems **10** containing B←N coordination from **9** by n-BuLi and Mes_2_BF (Mes_2_ = 2, 4, 6-Me_3_C_6_H_2_), whose mechanism involves the coordination between thiazole N and aryl B and consequent electrophilic attack of electron-deficient boron to the β-site of adjacent thiophene (Wakamiya et al., 2006). Recently, Liu et al. utilized this route to produce a stable electron-deficient unit **11**. By co-polymerizing with D or A units, they constructed a series of novel photovoltaic polymers with outstanding performances (Dou et al., 2015).

#### BX_3_/hindered base system

**Figure 4:**
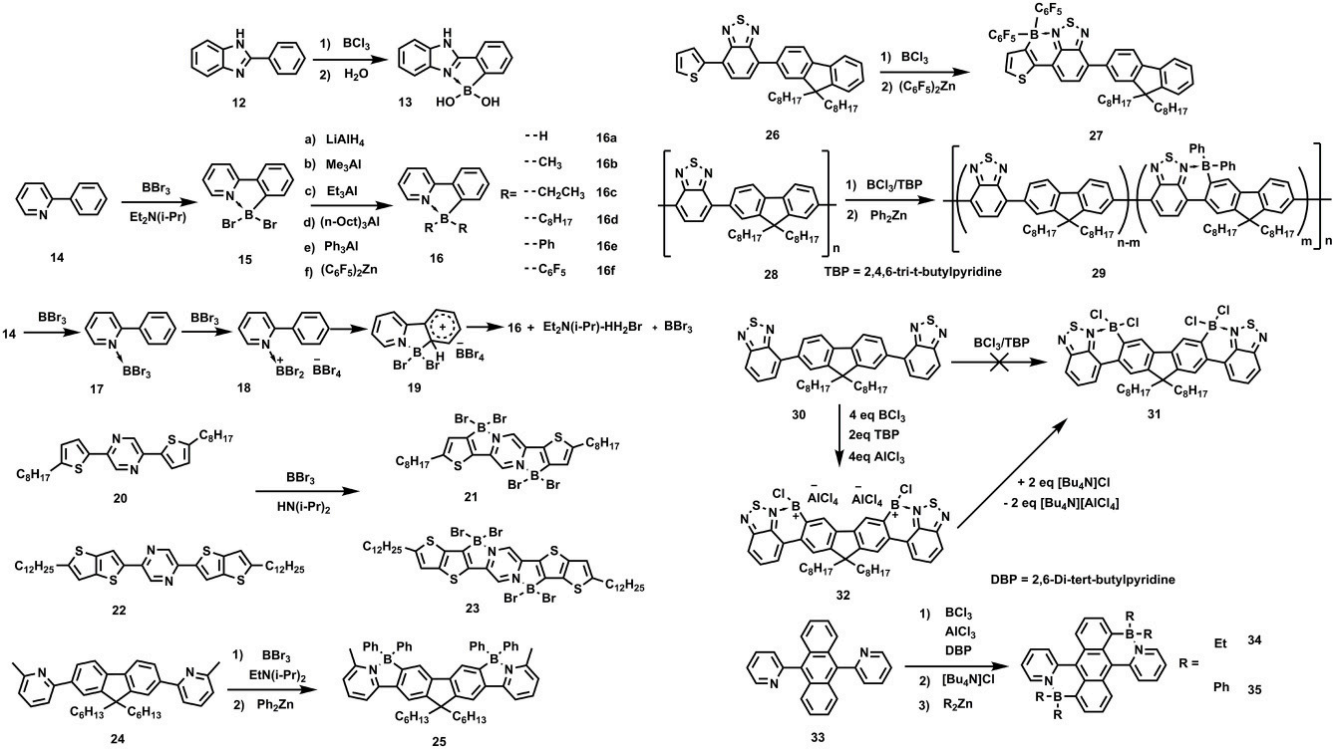
Examples of synthesis routes toward B←N embedded units involving BX_3_/ hindered base reaction system.

This synthetic method can be traced back to 1963, when Maclean et al. passed BCl_3_ into melts of 2-phenylbenzirnidazole (**12**) at 300°C and subsequently hydrolyzed in moisture air producing **13** (Letsinger and MacLean, 1963), as shown in Figure 4. The C-H borylation was considered to be reversible and the by-product HCl should be sequestered to improve the reaction yield. Accordingly, in 2010, Murakami et al. improved the method by adding a hindered base, Et_2_N(*i*-Pr) to absorb the protic by-product (Ishida et al., 2010). They used **14** as starting reactant, by adding 3 eq BBr_3_ and 1 eq Et_2_N(*i*-Pr) at 0°C, achieving **15**. **15** was not stable in moisture due to the electron-drawing property of Br, endowing B atom strong electrophilic. Further functionalization at B atom can be realized by adding organometallic reagents to substitute the Br atoms, affording a series of stable ladder-type π-units containing B←N bonds (**16a**-**16f**). The reaction mechanism was proposed as follow: the Lewis acid-base coordination between **14** and BBr_3_ provided **17**; then, another BBr_3_ captured a Br^−^ from **17**, leading to trivalent cationic boron species **18**; finally, the cationic boron attacked the neighboring aromatic unit, generating the circular **16**. After that, several conjugated units containing B←N coordination with tunable emission and aggregating-induced emission properties were synthesized by this method (Wong et al., 2012, 2016; Zhao et al., 2013). Recently, based on this strategy, fused π-electronic units with good co-planarity, red-shifted absorption, and depressed energy levels of **21**, **23**, and **25** have been synthesized from **20**, **22**, and **24**, respectively (Yusuf et al., 2016; Zhu et al., 2016; Li Y. et al., 2018). These π-electronic units containing B←N coordination are potentially useful to construct organic semiconductors for electronic device applications. As this strategy involves the electrophilic attack on the aromatic units, the electron-rich nature of the aromatic cycles is critical to the C-H borylation. Ingleson and Turner et al. employed **26** as precursor to conduct the C-H borylation (Crossley et al., 2015). It's found that the C-H borylation occurred on the thiophene rather than the fluorene, presumably due to the more electronic-rich nature of thiophene than fluorene, facilitating electrophilic attack on the thiophene. Not only the small molecules, but also the polymers can undergo this reaction. Ingleson and Turner et al. also applied this method to modify the copolymer **28**, yielding near-infrared emitting polymer **29** (Crossley et al., 2017).

Although the BX_3_/hindered base reaction condition has been demonstrated to be widely applicable to the C-H borylation, it's invalid in some cases. For example, Ingleson and Turner et al. found that precursor **30** can not afford **31** upon adding BX_3_, e.g., BCl_3_ or BBr_3_ and the hindered base, e.g., EtN(*i*-Pr)_2_ or 2,4,6-tri-t-butylpyridine (TBP) (Crossley et al., 2015). Otherwise, the C-H borylation occurred by adding excess BCl_3_ (ca. 4 eq), 2 eq of TBP and 4 eq of AlCl_3_, yielding intermediate **32**, which was readily transformed to **31** by adding Bu_4_NCl. The addition of 4 eq of AlCl_3_ was regarded to be essential to ensure the fully conversion to intermediate **32**. The function of AlCl_3_ herein is similar to its effect in the classic Friedel–Crafts reaction. It's worth to note although the structure units of **30** is the same to the repeating units of polymer **29**, the C-H borylation conditions were different for this two precursor, indicating the different reaction law in small molecules and polymers for C-H borylation. Recently, 2D conjugated units containing B←N coordination (**34** and **35**) were reported, also based on this method (Liu K. et al., 2017).

### Synthesis of π-electronic units containing B-N covalent bonds

#### Electrophilic cyclization between boron and aromatic units

**Figure 5:**
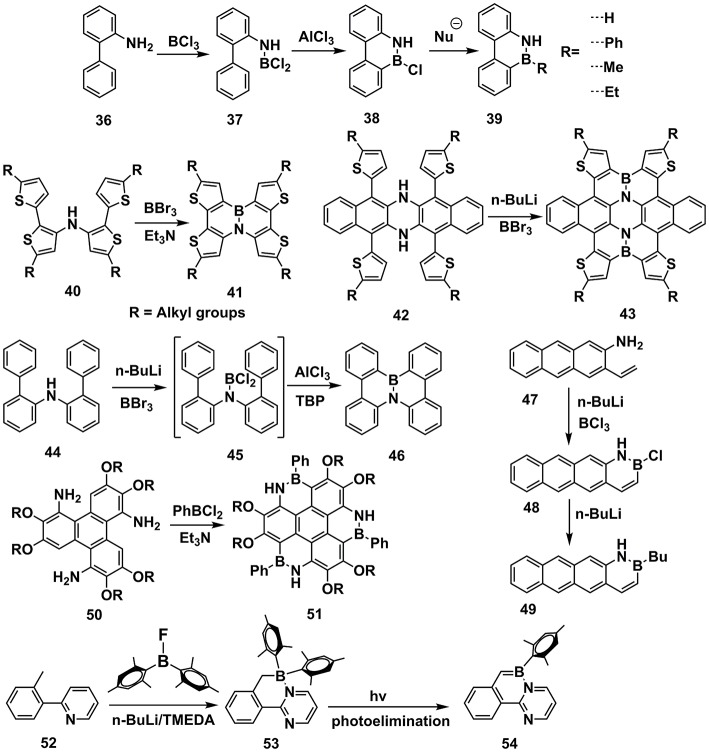
Examples of synthesis routes toward B-N embedded units via electrophilic cyclization between boron and aromatic units.

This method involves the Friedel-Crafts cyclization, in which BX_3_ and Lewis acid are usually required to complete the cyclization (Figure 5). In 1958, Dewar et al. initially conducted the synthesis work from **36** by adding BCl_3_ and AlCl_3_ to obtain 9,10-azaboraphenanthrenes (**38**) (Dewar et al., 1958). Further modification on the B atom led to a series of BN-substituted phenanthrene derivatives (**39**). Consequently, a family of B-N embedded PAHs was synthesized via the similar strategies (Dewar and Dietz, 1959; Chissick et al., 1960; Dewar and Poesche, 1963, 1964). In 2013, Pei and coworkers reported BN-substituted tetrathienonaphthalene derivatives (**41**) (Wang X. Y. et al., 2013), starting from **40** by adding BBr_3_ and Et_3_N. BBr_3_ attacked the imine and consequently electrophilic attacked the β-site of thiophene to finish the cyclization. Latterly, they revealed extended π-conjugated structure **43** with similar cyclization methods (Wang et al., 2014). It's worth to note that the conjugation of **42** is more extended than **40**, leading to weaker electron-rich of imine groups in **42**. Accordingly, the n-BuLi was required to facilitate the attack of BBr_3_ to imine in **43**. Similarly, starting from **44**, Nakamura et al. utilized n-BuLi and BBr_3_ to prepare the intermediate **45** (Hatakeyama et al., 2011). Due to weaker electron-rich properties of phenyl than thiophene, the Lewis acid such as AlCl_3_ and hindered base TBP were required to complete the electrophilic cyclization to produce **46**. Liu et al. reported the synthesis of B-N embedded tetracene **48** and **49**, starting from **47** (Ishibashi et al., 2014, 2017). Pei et al. also reported synthesis of heterocoronene (**51**) by adding PhBCl_2_ and Et_3_N to **50** and heated to 180°C in *o*-DCB (Wang et al., 2015c). Based on similar methods, ladder-type conjugated units substituted by B-N covalent bonds were also synthesized (Wang X. et al., 2013; Zhou et al., 2016). Wang et al. revealed an electrophilic cyclization between B and methyl located on phenyl, obtaining unsaturated **53**, which was subjected to photoelimination, leading to **54** (Lu et al., 2013; Ko et al., 2014; Yang et al., 2015, 2016, 2017).

#### Chelation of aromatic N and B precursor

**Figure 6:**
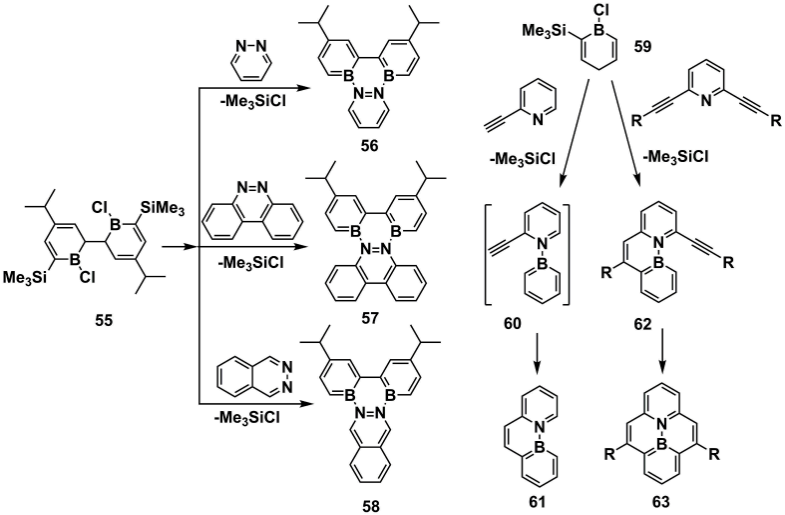
Examples of synthesis routes toward B-N embedded units via chelation of aromatic N and B precursor.

This method involves the chelation of B precursor and N Lewis base to eliminate a by-product (Figure 6). In 2003, Piers et al. reported the chelation of **55** with pyridazine and benzo[c]cinnoline, eliminating Me_3_SiCl to obtain **56** and **57**, respectively (Emslie et al., 2003). Latterly in 2006, this method was applied to synthesize **58** (Jaska et al., 2006). In 2007, they further revealed synthesis of **61** by chelation of **59** with 2-ethynyl-pyridine, leading to intermediate **60**, which experienced smooth cyclization without any catalyst (Bosdet et al., 2007a). In another aspect, the chelation of **59** with 2, 5-diethynyl-pyridine afforded **62**, which required catalytic amount of PdCl_2_ to complete the second cyclization of the ethynyl group to obtain the pyrene analog with internalized B-N substitution **63** (Bosdet et al., 2007b). The chelation and ethynyl-cyclization strategies were spread widely to prepare a series of PAHs embedded with B-N bonds (Jaska et al., 2007; Bosdet et al., 2010; Benedikt et al., 2013).

### Synthesis of π-electronic units containing N–B←N groups

**Figure 7:**
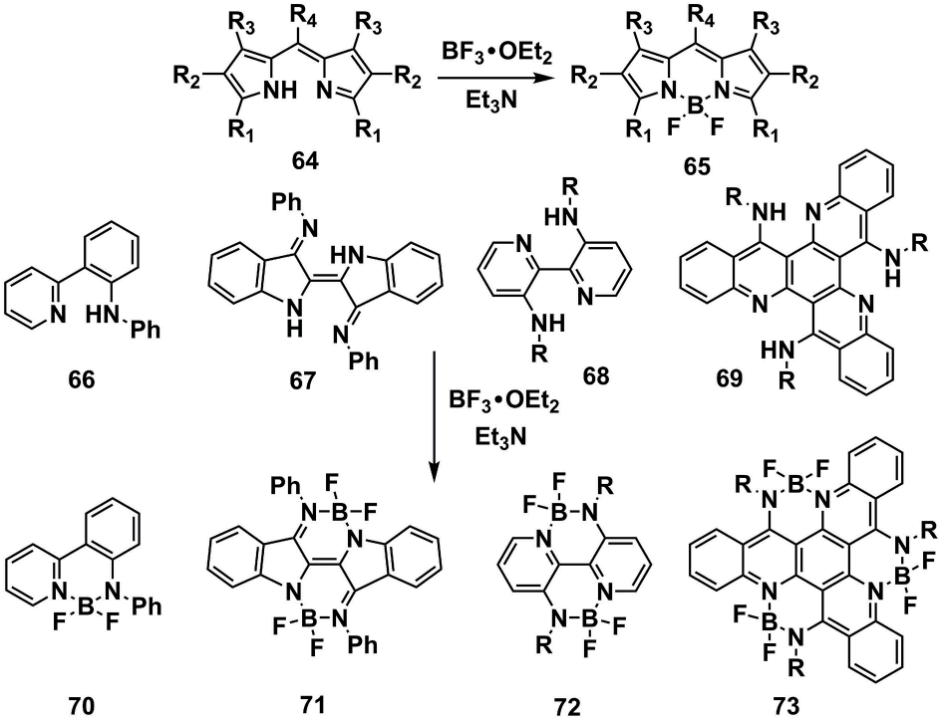
Examples of synthesis routes toward π-units containing N-B←N groups via BF_3_•OEt_2_/Et_3_N reaction condition.

A family of conjugated molecules containing N–B←N groups has been intensively explored as fluorescence dyes with high absorption coefficients and fluorescence quantum efficiency, which were widely utilized in OSCs, OLEDs, sensing, and imaging, etc (Li et al., 2013; Fu et al., 2015; Lin et al., 2015; Dou et al., 2017). The synthesis routes toward N–B←N groups usually require a precursor equipped with an amino group and an aromatic nitrogen at suitable position for chelation of the boron. BF_3_•OEt_2_/Et_3_N is most widely used reaction condition (Figure 7). Taking the typical dye boron dipyrromethene (BODIPY) as an example, the precursor **64**, usually synthesized from pyrrole derivatives and aldehydes, is readily to obtain the BODIPY skeleton **65** by adding BF_3_•OEt_2_/Et_3_N (Loudet and Burgess, 2007). This reaction condition is widely applicable to the precursors with the features of containing amino group and aromatic nitrogen atoms at appropriate positions, e.g., **66**, **67**, **68**, and **69** (Araneda et al., 2011; Nawn et al., 2013; Hao et al., 2014; Qiu et al., 2016).

## Optoelectronic properties and OPV applications

### π-electronic units containing B←N coordination bonds

**Figure 8:**
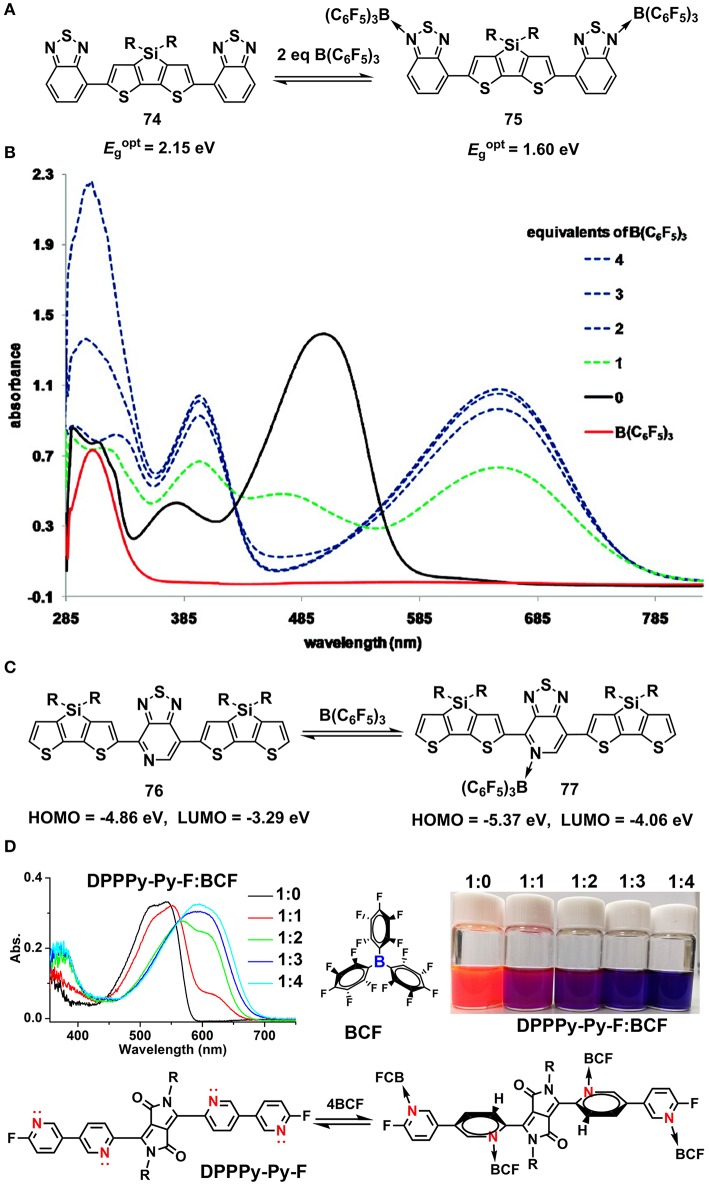
Lewis acid-base coordination **(A–C)** and the variation of UV-Vis absorption spectra of **74** upon coordinating with BCF **(B)**, Reprinted with permission from Welch et al. (2009). Copyright (2009) American Chemical Society. Manipulation of optical absorption spectra of DPP molecules upon coordinating with BCF **(D)**, Reprinted with permission from Huang et al. (2018). Copyright (2018) Elsevier Ltd.

In a long time, the inter-molecular coordination between B and N has been well-demonstrated to adjust the optoelectronic properties of conjugated molecules. It's well-known that the B atom is an electron-deficient center (Lewis acid) due to the existence of an unoccupied orbital while the N atom is an electron-rich center (Lewis base) owing to the existence of un-bonded pair of electron. As such, typical Lewis acid-base coordination between B and N atoms occurs when molecules containing N and B atoms are mixed together (Maria and Gal, 1985; Piers, 2005). It's has been revealed that the optoelectronic properties of conjugated molecules containing N atoms can be readily amendable when mixed with boride Lewis acid, e.g., BF_3_, BCl_3_, BBr_3_, and B(C_6_F_5_)_3_ (BCF). In 2009, Bazan and co-workers reported the bandgap control of benzothiadiazole-based oligomers via Lewis acid of B(C_6_F_5_)_3_ (Welch et al., 2009). As shown in Figures 8A,B, upon stoichiometric coordination with BCF, the absorption band of **74** red-shifted and the optical bandgap (*E*${}_{\text{g}}^{{}^{\circ}}$pt) decreased from 2.15 to 1.60 eV. In 2011, they further implemented the method to a series of oligomers and polymers. The HOMO and LUMO, estimated from ultraviolet photoelectron spectroscopy (UPS) were found to synergetic lowering due to the introduction of electron-deficient center B to the conjugated backbone (Figure 8C) (Welch and Bazan, 2011). It's interpreted that the Lewis acid BCF pulled the electron density away from the conjugated backbone, altering the electron topology and leading to decreased HOMO/LUMO and optical bandgap. In 2017, the strategy was employed to the dye molecules of 7-azaisoindigo, by using BF_3_ to amend the energy levels and optical absorption (Randell et al., 2017). Recently, we synthesized a series of pyridine end-capped diketopyrrolopyrrole (DPP) dye molecules and systematically explored the optical bandgap alteration upon coordinating with BCF (Huang et al., 2018). The effects of stoichiometry and equilibrium of the Lewis acid-base interactions on the optical bandgaps were studied (Figure 8D).

Lewis acid-base complexation of conjugated polymers containing aromatic N atoms with BCF can also adjust the performances of OFET and OLED devices. Heeney et al. synthesized two indenopyrazine-based copolymers, with which the OFET devices were prepared. They explored the effects of doping BCF to the copolymers on the device performances (Han et al., 2016). It's found that by doping the polymers with BCF in a certain amount, e.g., 0.075 equiv, the hole mobility can be increased up to 11-fold along with the reduced threshold voltages. Otherwise, increased the amount of BCF to a critical amount, the OFET performances would be adversely affected. It's deduced by the authors that moderate amount of BCF leads to effective traps filling and positive effects on the device operation while the excess amount of BCF gives rise to defect formation and structural disorder, which negatively affects the device performances. Bazan and Nguyen et al. studied the color turning of OLED by BCF doping (Zalar et al., 2012). They selected a fluorescent copolymer of fluorene and pyridine. By doping the polymer with BCF, the OLED emission color red-shifted obviously.

**Figure 9:**
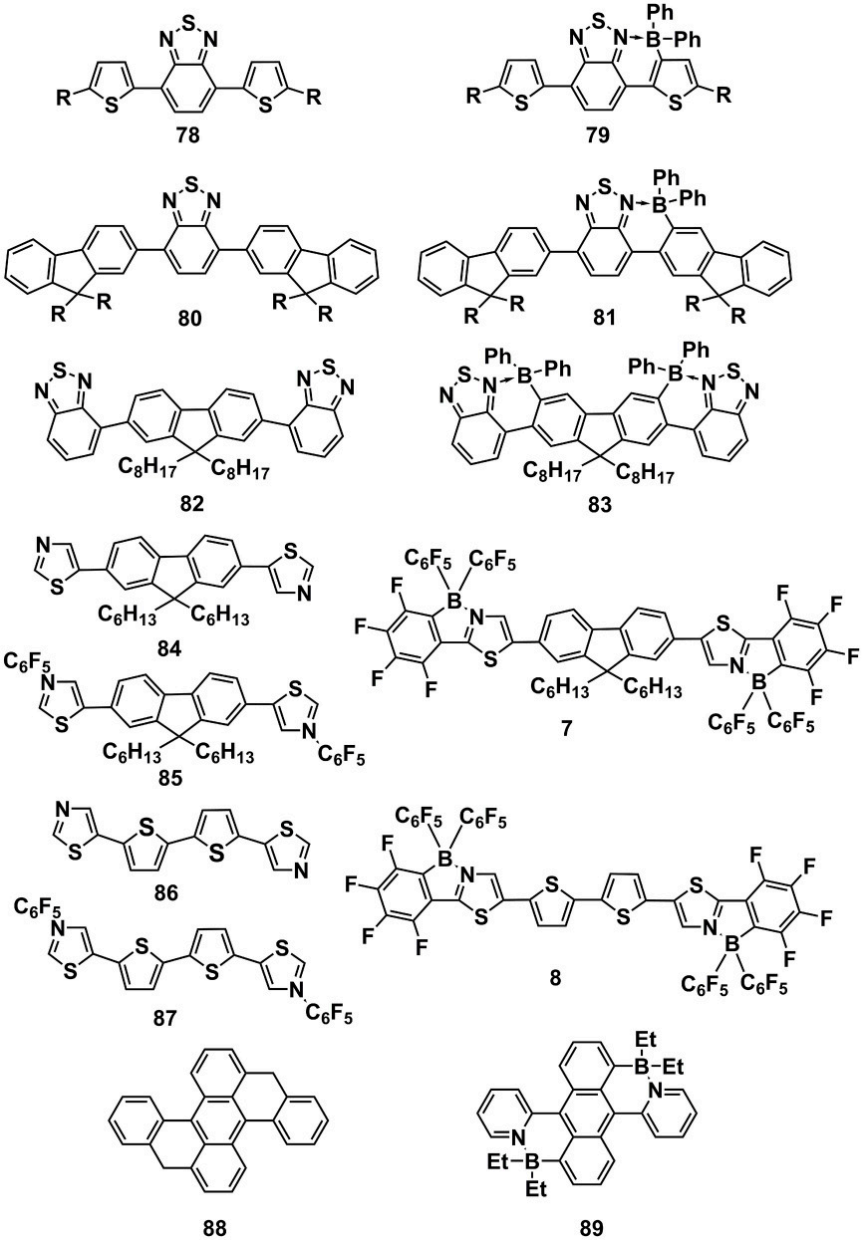
Molecular structures of B←N embedded π-units and their precursors before cyclization.

**Figure 10:**
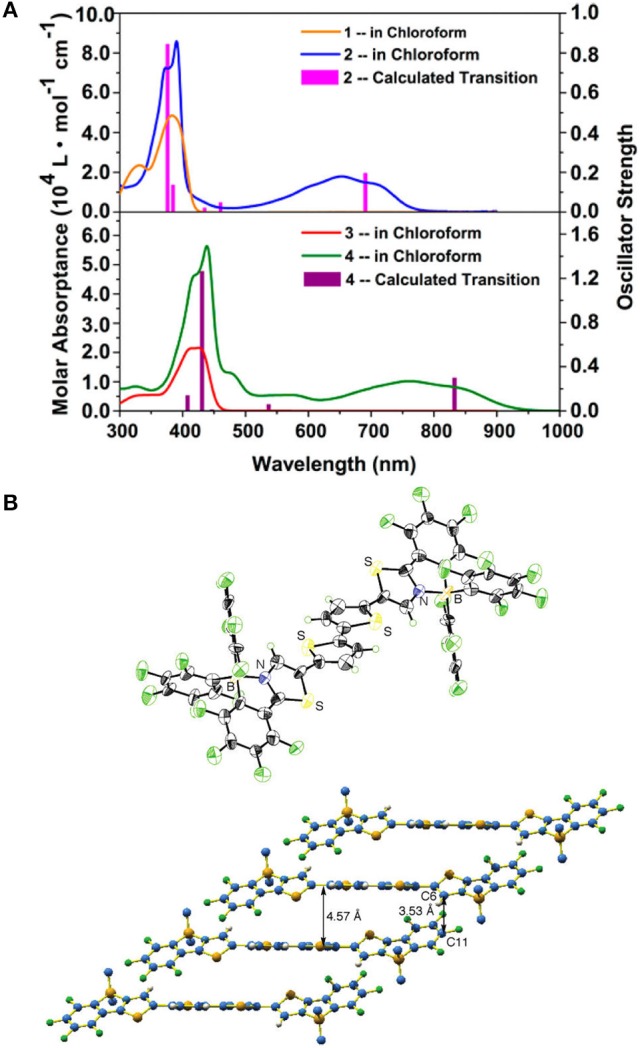
Absorption spectra of **20**/**21** (upper) and **22**/**23** (bottom) in chloroform **(A)** and Single crystal structure of **8 (B)**. Reprinted with permission from Job et al. (2010) and Zhu et al. (2016). Copyright (2010, 2016) American Chemical Society.

Although the inter-molecular B←N interactions are effective to adjust the optoelectronic properties of conjugated molecules, this method are not applicable for OPV devices because the inter-molecular B←N complexation is unstable and the boride molecule dopants may lead to defect formation and hinder the molecular order packing. Consequently, incorporating B←N bonds into the molecular skeletons are more feasible for OPV applications. Recent development of synthesis protocols promoted the birth of several π-units containing B←N bonds (Figure 9). As the frontier orbital energy levels, i.e., HOMOs and LUMOs and optical absorption are critical parameters for the PCEs of OPV devices, we summarized these parameters of some B←N embedded π-units, as shown in Table 1. For **20** and **22** (Figure 4), after introducing the BBr_2_ groups into the backbones, the LUMOs were decreased significantly by 0.96 and 0.53 eV, corresponding to **21** and **23**, respectively (Zhu et al., 2016). Moreover, remarkably red-shifted absorption band to near-infrared region occurred after introducing the BBr_2_ groups into backbones (Figure 10A). Similarly, LUMOs of **79**, **81**, and **83** also lowered obviously in comparison to their precursors of **78**, **80**, and **82** (Crossley et al., 2015). It's worth to note that the LUMO depressed remarkably with the slightly changed HOMOs, leading to decreased bandgaps. An interesting comparison from precursor **84** (**86**), to inter-molecular B←N complex **85** (**87**), and to cyclization product **7** (**8**) further demonstrates the outstanding ability of B←N unit to depress the LUMO energy levels of π-units (Job et al., 2010). Not only compared to their precursors before cyclization, but also in contrast to the all-carbon analogs, the B←N embedded π-units also exhibit significantly lowered LUMOs, as illustrated by **88** and **89** (Liu K. et al., 2017). These results indicate that the B←N embedded π-units usually have depressed LUMOs and expanded absorption bands in contrast to their precursors and all-carbon analogs due to the introduction of electron-deficient center B.

**Table 1:** Frontier orbital energy levels and optical bandgaps of B←N embedded π-units.

**Compounds**	**HOMO (eV)**	**LUMO (eV)**	$E_{g}^{opt}$ **(eV)*^b^***
**20**	−5.66	−3.44	–
**21**	−5.72	−4.40	1.59
**22**	−5.54	−3.68	–
**23**	−5.40	−4.21	1.34
**78**	−6.00	−3.73	2.29
**79**	−5.96	−4.15	1.73
**80**	−6.27	−3.52	2.59
**81**	−6.19	−4.11	1.92
**82**	−6.34	−3.52	2.82
**83**	−6.11	−4.11	2.02
**84**	−5.52*^a^*	−1.78*^a^*	3.58
**85**	−6.68*^a^*	−2.88*^a^*	3.52
**7**	−6.36*^a^*	−3.01*^a^*	3.11
**86**	−5.38*^a^*	−2.21*^a^*	3.25
**87**	−6.55*^a^*	−3.34*^a^*	3.19
**8**	−6.17*^a^*	−3.30*^a^*	2.87
**88**	−4.54*^a^*	−1.81*^a^*	–
**89**	−4.76*^a^*	−2.44*^a^*	–

a Obtained by theoretical calculations. Other HOMOs and LUMOs were estimated by electrochemistry method.

b *Calculated by 1240/λ_onset_. λ_onset_ is the absorption onset of UV-Vis absorption spectra*.

Other properties that essentially affect the PCEs of OPV devices are the solid packing order and charge carrier mobility. Single crystal data indicate the B←N embedded π-units, e.g., **8** also have good co-planarity, rigidity, and ordered π-π packing, as shown in Figure 10B (Job et al., 2010). It's beneficial to the charge transport in solid state, which is critical to the OPV performances. These features of depressed LUMOs, namely, strong electron-affinity and good molecular planarity and π-π packing order make the B←N embedded π-units suitable for the electron-transporting materials. For example, the dimeric B←N embedded CPT showed electron mobility of 1.5 × 10^−4^ cm^2^/V•s tested by time-off-light (TOF) carrier-mobility measurement (Wakamiya et al., 2006). Comprehensively, the B←N embedded π-units are excellent electron-deficient moieties with good co-planarity, depressed LUMOs, broadened absorption bands, and high electron mobility, which are promising for the application of photovoltaic materials, especially for acceptor materials. However, the contributions to exploit the potential of B←N embedded π-units for OPV application are scarcely revealed.

**Figure 11:**
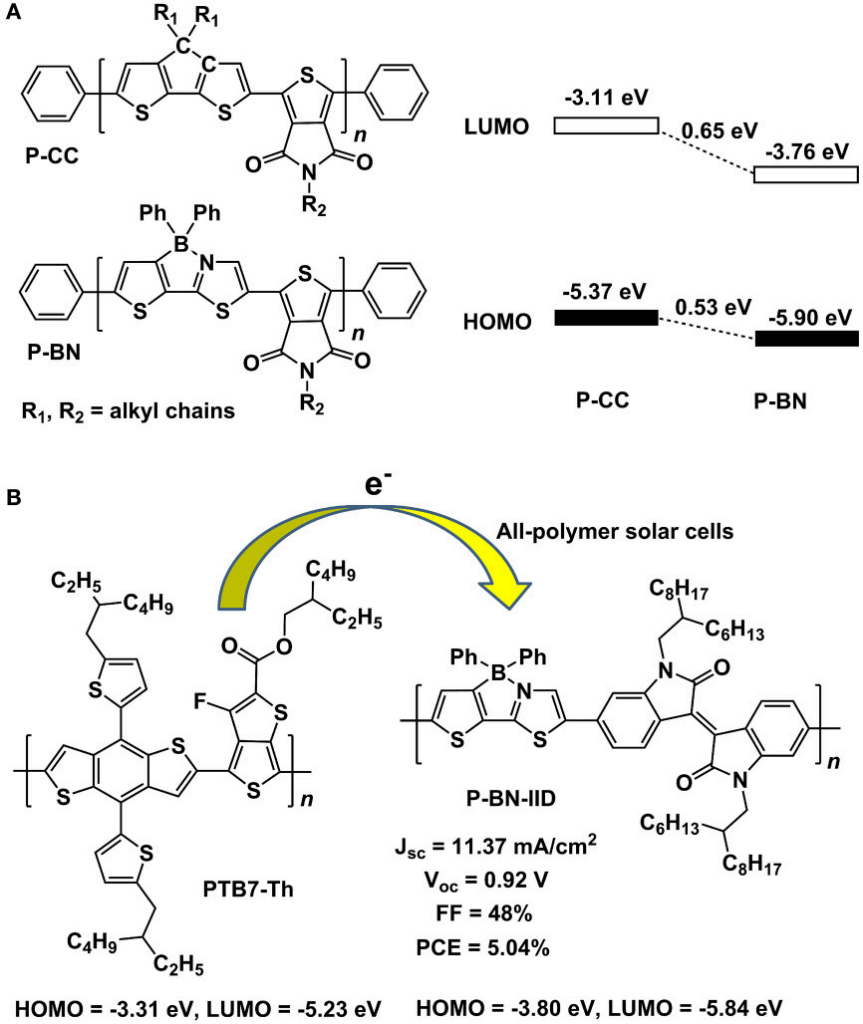
Molecular structures of P-CC and P-BN and the alignment of frontier orbital energy levels **(A)** and parameters of all-polymer solar cells based on PTB7-Th and P-BN-IID **(B)**.

Until recently, Liu and co-workers' pioneering work demonstrated the great potential of B←N embedded π-units for the construction of OPV materials. They selected B←N embedded CPT (BNCPT), which was developed in 2006 by Yamaguchi et al. (Wakamiya et al., 2006), as co-monomer to copolymerize with thieno[3,4-c]pyrrole-4,6-dione-1,3-diyl (TPD) unit, obtaining a novel conjugated polymer P-BN (Dou et al., 2015). For comparison, the all-carbon analog CPT was also copolymerized with TPD, leading to P-CC. The HOMO and LUMO of P-BN were significantly depressed by 0.65 and 0.53 eV, respectively, in contrast to the values of P-CC (Figure 11A), indicating the electron acceptor property of P-BN, which was further confirmed by the fluorescence quenching of P-BN with P3HT in solutions. These results demonstrated the B←N based copolymers are suitable for electron acceptor in OPV devices. Then, they synthesized another copolymer by combining BNCPT with isoindigo (IID), affording P-BN-IID with HOMO = −3.80 eV and LUMO = −5.84 eV (Zhao et al., 2016). Using PTB7-Th as electron donor, the all-polymer solar cells based on P-BN-IID exhibited a competitive PCE of 5.04% (Figure 11B). On the other hand, the BNCPT was also adopted to construct electron donor polymers by copolymerizing with its all-carbon analog CPT. This polymer displayed suitable HOMO and LUMO levels and exhibited a PCE of 3.74% by using PC71BM as electron acceptor (Zhang et al., 2015). These pioneering studies on the application of B←N based units to the OPV materials open a new window for the design of novel and highly efficient photovoltaic materials, not only for the polymers, but also for the small molecules.

### π-electronic units containing B-N bonds

**Figure 12:**
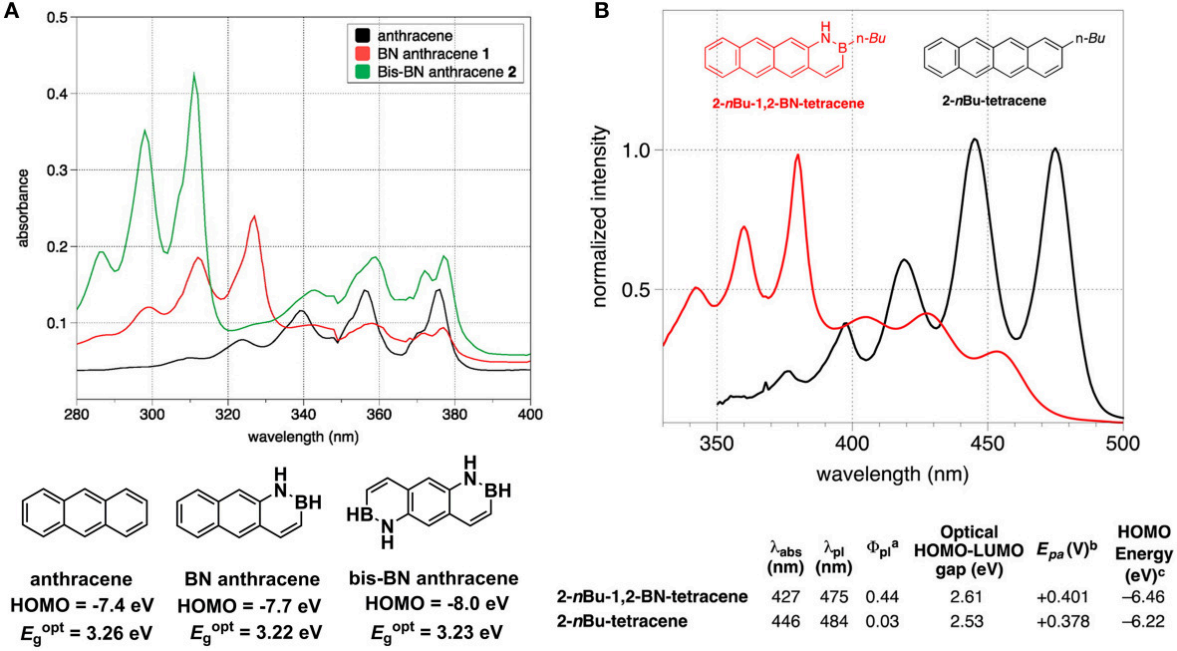
Absorption and energy levels of anthracene and B-N embedded anthracene **(A)** and tetracene and B-N embedded tetracene **(B)**. Reprinted with permission from Ishibashi et al. (2014, 2017). Copyright (2014, 2017) American Chemical Society.

As the B-N covalent bond is isosterism of C = C bond, replacing the C = C unit in a PAH with the isosteric B-N has emerged as a useful strategy to enlarge the library of π-conjugated units. The B-N embedded PAHs usually have similar geometric parameters but rather distinct electronic structures to its all-carbon analogs. As for the OPV materials, the energy levels, absorption spectra and solid state packing ability are extensively concerned. Herein, we summarized recent progress in some typical B-N embedded PAHs, emphasizing the comparison of B-N doped π-conjugated units to their all-carbon analogs in terms of frontier orbital energy levels, absorption spectra, bandgaps, as well as single crystal packing order. Liu et al. conducted systematic studies on the B-N embedded acenes, e.g., naphthalene, anthracene, and tetracene (Ishibashi et al., 2014, 2017; Liu Z. et al., 2017). In 2014, they revealed two B-N isosteres of anthracene, i.e., BN anthracene and bis-BN anthracene (Figure 12A) (Ishibashi et al., 2014). HOMO level tested from UV-photoelecton spectroscopy was −7.4, −7.7 eV, and −8.0 eV, respectively for anthrecene, BN anthracene, and bis-BN anthracene, indicating that the replacement of C = C with B-N gave rise to stabilized HOMO levels. Optical bandgaps estimated from the onset of the absorption spectra were similar for the three molecules. Comparing to anthracene, BN anthracene and bis-BN anthracene appeared a new absorption band at 310 nm, with relatively stronger oscillator strength, which mainly originated from the HOMO−1 to LUMO transition. Recently, they extended the reach of BN/CC isosterism to the tetracene, obtaining B-N perturbed tetracene (Figure 12B) (Ishibashi et al., 2017). In contrast to the all-carbon analog, the B-N perturbed tetracene showed slightly depressed HOMO and larger optical bandgap. Upon embedding B-N to tetracene, a blue-shift of HOMO to LUMO transition from 446 to 427 nm occurred and new absorption band originated from HOMO-1 to LUMO with stronger oscillator strength appeared around 380 nm. Very recently, they also disclosed that the orientation and location of B-N in the naphthalene exerted critical influence on the energy levels, bandgaps and absorption properties (Liu Z. et al., 2017).

**Figure 13:**
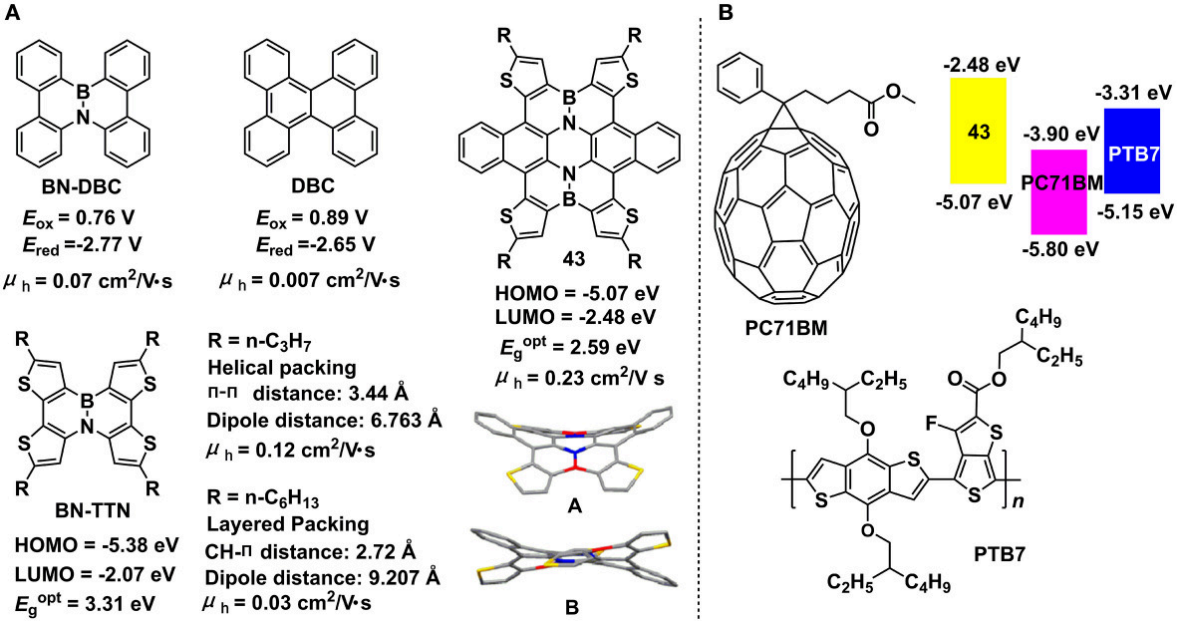
Molecular structures, energy levels, and single crystal parameters for B-N embedded 2D π-conjugated units **(A)** and photovoltaic applications of compound 43 as an electron donor or additive **(B)**. Reprinted with permission from Wang et al. (2014). Copyright (2014) American Chemical Society.

The B-N embedded dibenzo[g,p]chrysene (BN-DBC) reported by Nakamura et al. showed negative-shifted redox potential in comparison to the all-carbon analog, dibenzo[g,p]chrysene (DCB) (Figure 13A) (Hatakeyama et al., 2011), indicating the synergistic depression of HOMO and LUMO levels and unchanged electrochemical bandgaps. X-ray crystallography data revealed the twisted conformations and offset face-to-face stacking style with π-π distances of 3.3–3.6 Å for both DCB and BN-DCB. Although the similar molecular stacking style for DCB and BN-DCB, the hole mobilities were distinct, with 0.07 and 0.007 cm^2^/V•s, respectively for BN-DCB and DCB. The favorable hole mobility of BN-DCB are beneficial from the introduction of polar B-N unit into backbone leading to stronger electronic coupling between neighboring molecules. Pei and co-workers developed two BN-substituted tetrathienonaphthalene derivatives, i.e., BN-TTN-C3 and BN-TTN-C6 (Wang X. et al., 2013). Because of the different side chains, the two BN embedded units exhibited distinct packing mode, with helical and layered packing style for BN-TTN-C3 and BN-TTN-C6, respectively. BN-TTN-C3 displayed close π-π stacking (3.44 Å) in crystal state whereas BN-TTN-C6 showed CH-π interaction. Moreover, BN-TTN-C3 gave closer dipole-dipole interaction (6.763 Å) compared to that of BN-TTN-C6 (9.207 Å). Due to the higher ordered molecular packing, BN-TTN-C3 exhibited superior hole mobility of 0.12 cm^2^/V•s, elevated by one magnitude than the value of BN-TTN-C3 (0.03 cm^2^/V•s). Recently, they copolymerized the BN-TTN with thiophene units to afford the conjugated polymers with lowered HOMO levels (−5.46 ~ −5.67 eV) and strong intermolecular interactions (Wang et al., 2015b). OFET devices prepared from these azaborine-based polymers exhibited a champion hole mobility of 0.38 cm^2^/V•s. By changing the co-monomers, a vast of novel copolymers based on this azaborine unit can be obtained, predicting a great potential of this unit for electronic device applications.

Furthermore, they developed a straightforward strategy to produce the largest BN embedded heteroaromatic (**43**) to date (Wang et al., 2014). Single crystal X-ray diffraction indicated the significant distorted conformation of **43** due to the steric hindrance among peripheral rings and two different conformations (A and B) were found in the same crystal. The single crystal showed a columnar stacking style along the 011 direction and one-dimensional micro-ribbons can be obtained feasibly due to the strong π-π interactions. OFET devices based on the micro-ribbons gave a hole mobility of 0.23 cm^2^/V•s, a low threshold of −3 V, and a current on/off ratio of > 10^4^. Theoretical calculations indicated a depressed HOMO and unchanged LUMO of **43** in contrast to its all-carbon analog. HOMO estimated from electrochemistry and optical bandgap calculated from the absorption onset was −5.07 and −2.59 eV, respectively for **43**. OPV devices prepared with **43** as donor and PC71BM as acceptor gave a PCE of 3.12% and a *V*_oc_ of 0.96 eV (Zhong et al., 2016). Moreover, when it was added to PTB7/PC71BM system as additive, the ternary solar cells displayed improved PCE (4.75%) in comparison to the PCE of binary devices (3.91%) (Figure 13B). This is the first example of applying B-N embedded heteroaromatics to the OPV devices.

From the aforementioned discussion on the B-N embedded units, the following conclusions can be deduced. Different from the B←N bond, which amends the energy levels and optical absorption of π-units significantly, the B-N embedded π-units usually have similar or slightly different energy levels and optical bandgaps to their all-carbon analogs. Most of the B-N embedded units have large optical bandgaps and narrow absorption bands. Extending the conjugation of the B-N embedded π-units would broaden the absorption bands. Introducing the dipolar B-N bond into the conjugated backbone would enhance the inter-molecular interaction, facilitating the ordered π-π stacking and enhancing the hole mobility in contrast to their all-carbon analogs. Accordingly, B-N embedded π-units are excellent electron-rich units and promising candidates for construction of OPV materials. Otherwise, to now, OPV applications involving the B-N embedded π-units are scarcely revealed. From my point of view, these B-N embedded PAHs are promising for OPV applications and will represent an important direction of OPV materials.

### π-electronic units containing N–B←N groups

**Figure 14:**
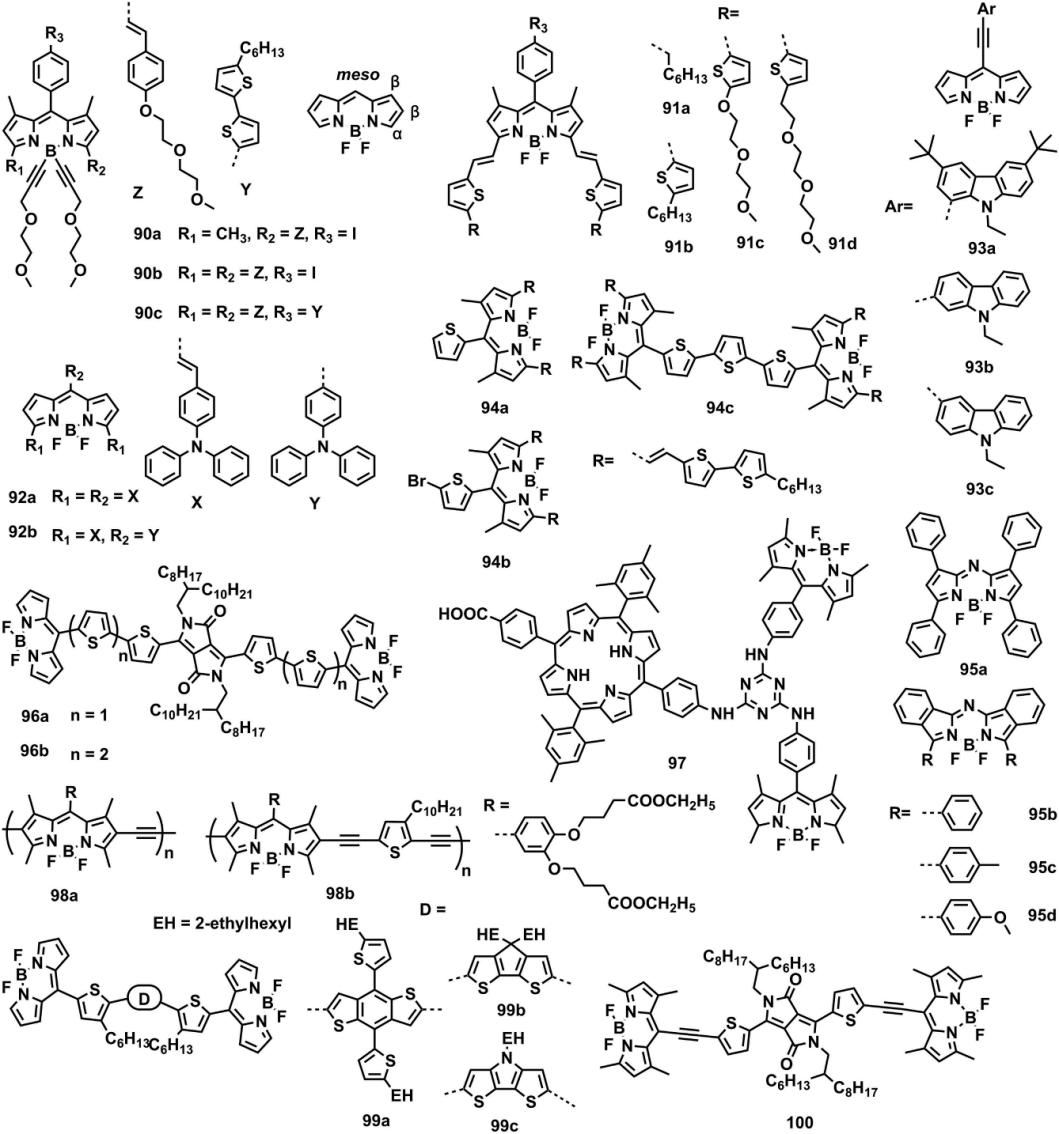
Molecular structures of BODIPY-based photovoltaic materials.

The typical π-electronic unit containing N–B←N group is the 4,4′-difluoro-4-bora-3a,4a-diaza-s-indacene (BODIPY), possessing unique optoelectronic properties, e.g., strong molar extinct coefficient (10^5^ M^−1^•cm^−1^), low-lying HOMO (−5.5 eV) and LUMO (−3.5 eV), strong electron affinity, and high fluorescence quantum yield, which has drawn much attention in the field of labeling and chemical sensors (Sekiya et al., 2009; Lu et al., 2014). For OPVs, the BODIPY also plays an important role, either in the electron donor or acceptor materials. In general, α, β, and *meso*-positions are readily available for chemical modification to adjust the properties of BODIPY for OPV applications (Figure 14). Table 2 summarizes the optoelectronic and photovoltaic parameters for BODIPY-based molecules. In 2009, Roncali et al. initially reported the BODIPY-based small molecules modified at α-position with styryl and *meso*-position with iodobenzene, i.e., **90a** and **90b** as electron donor materials, affording an optimum PCE of 1.17 and 1.34%, respectively, by using PC61BM as electron acceptor material (Rousseau et al., 2009a). Interestingly, ternary device prepared by blending **90a**, **90b**, and PC61BM as active layer showed a promoted PCE of 1.70% (Rousseau et al., 2009b). Then, the further modification of **90b** at *meso*-position with oligothiphene gave **90c**, leading to an improved PCE of 2.17% due to the enhanced hole mobility (Rousseau et al., 2010). In 2012, Ziessle et al. substituted the α-position of BODIPY with vinylthiophene to obtain **91a**, **91b**, **91c**, and **91d**, exhibiting a maximum PCE of 1.40, 4.70, 0.90, and 1.50%, respectively (Bura et al., 2012). The highest PCE for **91b** was interpreted by its depressed HOMO levels, broad and strong external quantum response and high hole mobility. Replacing at α and *meso*-positions with triphenylamine produced **92a** and **92b**, giving a moderate PCE of 1.50 and 0.51%, respectively (Kolemen et al., 2014). However, tailoring the *meso*-substituents with carbazole units along with device technique optimization by thermal annealing and solvent vapor annealing, **93a**, **93b**, and **93c** yielded a superior PCE of 5.05, 3.99, and 4.80%, respectively, by using PC71BM as electron acceptor material (Jadhav et al., 2015). Zhan and coworkers synthesized the dimeric BODIPY bridged with oligothiophene at the *meso*-positions (**94c**) (Liu et al., 2014). Compared with the single BODIPY cores **94a** and **94b**, the dimeric molecule **94c** showed improved packing order when blended with PC71BM and enhanced hole mobility, leading to a higher PCE of 3.13%. Mueller et al. revealed the BODIPY analogs of **95a** and **95b**, giving a PCE of 1.2 and 1.1% by using C60 as electron acceptor to prepare the vacuum-processed solar cells (Mueller et al., 2012). Similarly, Kraner et al. studied the influence of side groups on the OPV performance of BODIPY analogs, **95b**, **95c**, and **95d** (Kraner et al., 2015).

**Table 2:** Optoelectronic and photovoltaic parameters for BODIPY-based materials.

**BNBP**	**HOMO/LUMO(eV)**	$E_{g}^{opt}$***(eV)***	**Active layers**	***V*_oc_ (V)**	***J_sc_(mA/cm^2^)***	**FF(%)**	**PCE(%)**
90a	−5.69/−3.66	1.95	90a/PC61BM	0.80	4.43	34	1.17
90b	−5.56/−3.75	1.70	90b/PC61BM	0.75	4.14	44	1.34
90c	−5.61/−3.80	1.70	90c/PC61BM	0.75	7.00	38	2.17
91a	−5.46/−3.81	1.6	91a/PC61BM	0.76	5.84	31	1.40
91b	−5.34/−3.84	1.45	91b/PC61BM	0.70	14.3	47	4.70
91c	−5.30/−3.85	1.42	91c/PC61BM	0.56	5.10	30	0.90
91d	−5.32/−3.86	1.48	91d/PC61BM	0.55	8.50	32	1.50
92a	−5.00/−3.59	1.41	92a/PC61BM	0.68	7.00	31	1.50
92b	−4.96/−3.42	1.54	92b/PC61BM	0.43	3.59	32	0.51
93a	−5.48/−3.44	1.72	93a/PC71BM	0.90	10.2	55	5.05
93b	−5.62/−3.42	1.88	93b/PC71BM	0.90	9.24	48	3.99
93c	−5.54/−3.46	1.85	93c/PC71BM	0.96	9.64	52	4.80
94a	−5.02/−3.64	1.40	94a/PC71BM	0.67	6.80	34	1.56
94b	−5.06/−3.74	1.38	94b/PC71BM	0.72	7.62	36	1.96
94c	−5.06/−3.73	1.41	94c/PC71BM	0.74	11.28	38	3.13
95a	−5.68/−4.01	1.62	95a/C60	0.96	3.15	48	1.20
95b	−5.22/−3.65	1.48/(1.55)	95b/C60	0.65/(0.81)	2.40/(8.0)	65/(59)	1.1/(3.8)
95c	−5.27/−3.63	1.55	95c/C60	0.71	7.00	55	2.7
95d	−5.10/−3.60	1.52	95d/C60	0.61	5.40	52	1.7
96a	−5.13/−3.50	1.71	96a/PC71BM	0.71	3.39	27	0.65
96b	−5.10/−3.40	1.67	96b/PC71BM	0.53	4.55	26	0.64
97	−5.62/−3.52	1.84	97/PCBM	0.90	10.48	56	5.29
98a	−5.58/−3.73	1.61	98a/PC61BM	0.76	4.00	43	1.3
98b	−5.45/−3.71	1.65	98b/PC61BM	0.80	4.82	51	2.0
99a	−5.40/−3.79	1.73	P3HT/99a	0.65	3.09	0.60	1.21
99b	−5.16/−3.82	1.54	P3HT/99b	0.62	3.90	0.63	1.51
99c	−5.14/−3.74	1.47	P3HT/99c	0.57	3.28	0.63	1.18
100	−5.36/−3.79	1.50	PTB7-Th/*p*-DTS(FBTTh_2_)_2_/100	0.76	7.19	0.53	2.84

In order to further expand the absorption band of BODIPY, covalently combining it with other dye molecules emerged as an effective strategy. For example, the DPP unit was introduced to link with BODIPY, resulting into **96a** and **96b** (Cortizo-Lacalle et al., 2014). By using PC71BM as the acceptor, **96a** and **96b** showed a moderate PCE of 0.65 and 0.64%, respectively, due to the over-strong aggregating ability of these dye molecules, leading to poor film morphology. The porphyrin moiety was also selected to connect with BODIPY, producing **97**, which exhibited a competitive PCE of 5.29% (Sharma et al., 2015). Moreover, the BODIPY was also utilized as building block for construction of conjugated polymers. By copolymerizing with acetylene and thiophene, conjugated copolymers of **98a** and **98b** can be obtained, showing low bandgap of 1.61 and 1.65 eV, respectively. By using PC61BM as acceptor, moderated PCEs around 2.0% can be obtained (Kim et al., 2010).

Except for using as donor materials, the BODIPY-based molecules are also qualified for the acceptor materials due to its strong electron affinity. BODIPY dimers bridged with BDT (**99a**), CPDT (**99b**), and DTP (**99c**) were synthesized to be used as electron-acceptor materials. By selecting P3HT as electron-donor material, fullerene-free devices based on these BODIPY dimers showed PCEs from 1.18 to 1.51% (Poe et al., 2014). Zhan et al. reported the DPP bridged BODIPY dimers (**100**), exhibiting an competitive PCE of 2.84% by using PTB7-Th/*p*-DTS(FBTTh_2_)_2_ (0.5:0.5) as donor (Liu W. et al., 2017).

**Figure 15:**
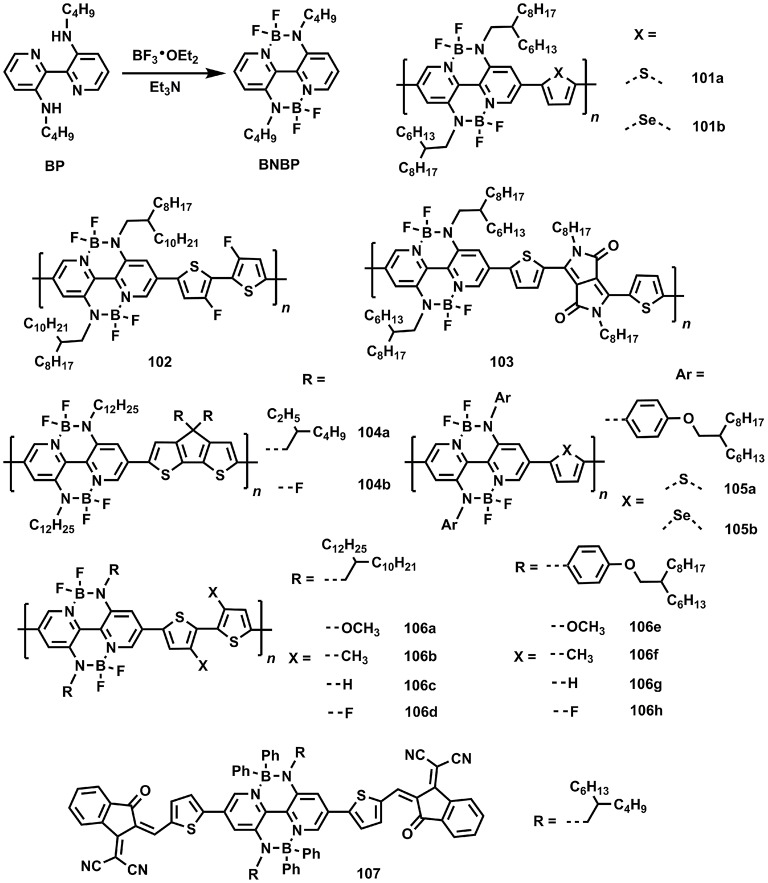
Molecular structures of BNBP-based photovoltaic materials.

Recently, a novel π-electronic unit containing N-B←N group, namely BNBP, with low-lying LUMO, bathochromic absorption, good co-planarity, and strong π-π interaction has been developed by Liu and coworkers. Owing to its strong electron-affinity, it's suitable to build conjugated copolymers for acceptor materials (Figure 15). Table 3 summarizes the optoelectronic and photovoltaic parameters for BNBP-based materials. The primary attempt to copolymerize with thiophene produced a typical D-A copolymer **101a**, showing the HOMO and LUMO of −5.77 and −3.50 eV, respectively. By selecting PTB7 as donor materials, all-polymer solar cells were fabricated, affording a high *V*_oc_ of 1.09 V and an impressive PCE of 3.38% (Dou et al., 2016). The high *V*_oc_ was mainly originated from the large offset between HOMO of PTB7 and LUMO of **101a**. By selecting PCDTBT, a donor polymer with low-lying HOMO of −5.42 eV, to prepare all-polymer solar cells with **101a**, a recorded *V*_oc_ of 1.3 V can be obtained (Ding et al., 2017). Using small molecule donor *p*-DTS(FBTTh_2_)_2_ to match with **101a** also gave a *V*_oc_ of 1.08 V and a PCE of 3.5% (Zhang Z. et al., 2017). Replacing the co-monomer from thiophene to selenophene led to the copolymer **101b**, which displayed depressed HOMO (−5.77 eV) and LUMO (−3.66 eV) compared to the values of **101a**. By using PTB7-Th as donor material, the all-polymer solar cells gave an improved PCE of 4.26%, which were interpreted by the enhanced driving force for the charge dissociation between PTB7-Th and **101b**, resulting from the deeper LUMO of **101b** in contrast to the value of **101a** (Ding et al., 2016). Furthermore, when the 3,3′-difluoro-2,2′-bithiophene (fBT) unit was utilized to copolymerize with BNBP, the resulting polymer **102** exhibited a high electron mobility of 2.4 × 10^−4^ cm^2^/V•s due to the intra-molecular F…S interaction that locked the conformation, enhanced the co-planarity, and facilitate the ordered molecular packing. Therefore, all-polymer solar cells based on PTB7-Th/**102** afforded a recorded PCE of 6.26% for N-B←N based acceptor materials (Long et al., 2016a). In order to further broadened the absorption band to lower energy, the DPP unit was selected as co-monomer to obtain copolymer **103**, showing a small optical bandgap of 1.56 eV and high electron mobility of 2.1 × 10^−4^ cm^2^/V•s. All-polymer solar cells based on PTB7/**103** gave a PCE of 2.69% (Long et al., 2016b).

**Table 3:** Optoelectronic and photovoltaic parameters for BNBP-based materials.

**BNBP**	**HOMO/LUMO(eV)**	$E_{g}^{opt}$ ***(eV)***	**Active layers**	***V*_oc_ (V)**	***J_sc_(mA/cm^2^)***	**FF(%)**	**PCE(%)**
101a	−5.77/−3.50	1.92	PTB7/101a	1.09	7.09	44	3.38
101b	−5.84/−3.66	1.87	PTB7-Th/101b	1.03	10.02	42	4.26
102	−5.87/−3.62	1.86	PTB7-Th/102	1.07	12.69	47	6.26
103	−5.45/−3.87	1.56	PTB7/103	0.88	7.54	41	2.69
104a	−5.64/−3.45	1.85	P3HT/104a	1.01	4.98	35	1.76
104b	−5.89/−3.60	1.82	PTB7-Th/104b	0.99	8.78	44	3.76
105a	−5.74/−3.72	1.97	PTB7-Th/105a	1.11	7.58	45	3.77
105b	−5.75/−3.73	1.87	PTB7-Th/105b	1.07	9.21	45	4.46
106a	−5.37/−3.39	1.72	106a/PC71BM	0.70	2.02	41	0.59
106b	−5.81/−3.32	1.83	–	–	–	–	–
106c	−5.92/−3.43	1.91	–	–	–	–	–
106d	−6.04/−3.62	1.90	PTB7-Ph/106d	1.09	10.13	47	5.16
106e	−5.46/−3.48	1.66	106e/PC71BM	0.76	7.59	51	2.92
106f	−5.84/−3.42	1.81	–	–	–	–	–
106g	−5.77/−3.51	1.85	–	–	–	–	–
106h	−5.85/−3.71	1.84	PTB7-Ph/106h	1.12	7.33	45	3.70
107	−5.34/−3.93	1.40	PTB7-Th/107	0.78	14.62	62	7.06

Additionally, the electron-rich CPT unit was also copolymerized with BNBP leading to **104a**, showing the HOMO and LUMO of −5.64 and −3.45 eV, respectively, which was matched well with the energy levels of P3HT (HOMO/LUMO = −5.20/−3.20 eV). As such, all-polymer solar cells based on P3HT/**104a** were prepared, affording a moderate PCE of 1.76% with a high *V*_oc_ of 1.01 V (Long et al., 2017a). When the alkyl groups on the CPT were replaced by F atoms, a new electron-rich unit, namely, 4,4-Difluoro-4H-cyclopenta[2,1-b:3,4-b′]dithiophene (fCPT) can be obtained. By copolymerizing with BNBP, the novel polymer **104b** exhibited depressed HOMO and LUMO in comparison with the values of **104a**. PTB7-Th was selected as donor material to afford a competitive PCE of 3.76% (Zhao et al., 2017b). Moreover, conjugated side groups were introduced to BNBP unit and copolymer **105a** and **105b** were produced. The conjugated side chains were considered to improve the electron mobility and a good PCE of 3.77 and 4.46% were demonstrated for **105a** and **105b**, respectively (Zhao et al., 2017a). Recently, they revealed an effective method to finely tune the HOMO and LUMO of BNBP-based polymers by varying the side chains, as evident by **106a**—**106h**, whose LUMOs were decreased gradually. **106a** and **106e** were suitable for the donor materials due to their high-lying energy levels whereas **106d** and **106h** were applicable for acceptor materials owing to their low-lying energy levels (Long et al., 2017b).

In general, these BNBP-based copolymers are suitable for acceptor materials due to the strong electron-affinity properties. Although several polymers based on BNBP were reported for OPV applications, small molecules related to this unit are scarcely revealed to now. Very recently, a small-molecule acceptor (**107**) built by end-capping BNBP with IC unit was reported, exhibiting a low-lying LUMO of −3.93 eV and wide absorption band (Liu F. et al., 2017). By selecting PTB7-Th as donor material, OPV devices gave a *J*_sc_ of 14.62 mA/cm^2^, a *V*_oc_ of 0.78 V, an FF of 62%, and a competitive PCE of 7.06%. This attempt points out the great potential of BNBP as a building block for small-molecule acceptor materials. We anticipate that the small molecules based on BNBP and its derivatives are also interesting and promising for non-fullerene acceptor materials. This field is blank to now and we strongly perceive that the small molecules based on novel π-electronic units containing N-B←N groups for OPV applications leave a large space and will be a research hot drawing great attention.

## Prospect

In summary, the OPV applications of BN embedded π-conjugated electronic units are in infancy. From our point, the following aspects will presumably be the potential research interests concerning the BN perturbed π-conjugated units.

For B-N covalent bond embedded π-conjugated units, they are suitable to act as electron-rich units to construct electron-donor materials owing to their high-lying energy levels, good backbone co-planarity, and high hole mobility. However, their absorption bands should be further broadened to lower energy (600–800 nm) to enhance the light harvesting ability. On the one hand, novel π-units containing B-N bond with extended conjugation should be developed to further red-shift the absorption bands. On the other hand, linking the B-N embedded π-conjugated units with low bandgap electron-deficient units, e.g., PDI, NDI, DPP, IID, IC, TPD, and BT can be an effective method to decrease the optical bandgaps and shift the absorption bands to longer wavelength. To this end, fine-tuning the energy levels and optical absorption via D-A combination of B-N embedded π-units and electron-deficient units will be a systematical job.

For B←N and N-B←N embedded π-units, the low-lying energy levels, red-shifted absorption bands, and good electron mobility make them promising electron-deficient units for construction of acceptor materials. To now, most studies have been focusing on the BODIPY-based donor materials and a few novel units containing B←N or N-B←N, e.g., BNCTP and BNBP were used to construct polymer acceptor materials. However, the currently revealed structures are still limited in terms of electron affinity, conjugation degree and light absorption. Great research space remains in the development of novel fused π-units containing B←N or N-B←N groups with depressed energy levels and strong light-harvesting ability. Upon judicious molecular tailoring, these B←N or N-B←N embedded π-units possess high electron affinity and good rigidity, which may be comparable to those typical electron-deficient units, e.g., PDI, NDI, DPP, and IID. As such, small-molecule acceptors established with B←N or N-B←N embedded π-units will presumably be an important family of OPV materials, although few examples has been reported to now.

Challenges also exist in these structures, from synthesis routes, to material stability, and processability. The synthesis routes usually involve the usage of BX_3_, which is strong Lewis acid, toxic, and easily subjected to hydrolysis. Similarly, some of the BN embedded units are unstable in moisture and cannot be purified by typical chromatographic methods due to the Lewis acid property of B atom (Crossley et al., 2015; Zhu et al., 2016). Moreover, how to introduce bulk substituents into the backbone to ensure sufficient solution processability without sacrificing the molecular co-planarity and packing order is also a challenge. These challenges should be fully taken into account when designing BN embedded units for OPV applications. However, we expect that BN embedded units will draw great attention for the construction of OPV materials.

## Author contributions

JH conceived, designed, and wrote the manuscript. YL retrieved the literature and edited sections of the manuscript. All authors approved it for publication.

### Conflict of interest statement

The authors declare that the research was conducted in the absence of any commercial or financial relationships that could be construed as a potential conflict of interest.
